# Plant-Derived Exosomes: Carriers and Cargo of Natural Bioactive Compounds: Emerging Functions and Applications in Human Health

**DOI:** 10.3390/nano15131005

**Published:** 2025-06-30

**Authors:** Sorur Yazdanpanah, Silvia Romano, Anna Valentino, Umberto Galderisi, Gianfranco Peluso, Anna Calarco

**Affiliations:** 1 Department of Experimental Medicine, University of Campania “Luigi Vanvitelli”, Via Santa Maria di Costantinopoli 16, 80138 Naples, Italy; soruryazdanpanah@cnr.it (S.Y.); silvia.romano@iret.cnr.it (S.R.); umberto.galderisi@unicampania.it (U.G.); 2 Research Institute on Terrestrial Ecosystems (IRET), National Research Council of Italy (CNR), Via Pietro Castellino 111, 80131 Naples, Italy; gianfranco.peluso@unicamillus.org (G.P.); anna.calarco@cnr.it (A.C.); 3 National Biodiversity Future Center (NBFC), 90133 Palermo, Italy; 4 Faculty of Medicine and Surgery, Saint Camillus International University of Health Sciences, Via di Sant’Alessandro 8, 00131 Rome, Italy

**Keywords:** plant-derived exosomes, carrier, cargo, bioactive molecules, human health

## Abstract

Extracellular vesicles (EVs) have gained increasing attention in recent years as a valuable focus of scientific investigation, owing to their potential therapeutic properties and wide-ranging uses in medicine. EVs are a heterogeneous population of membrane-enclosed vesicles with lipid bilayers, released by cells from both animal and plant origins. These widespread vesicles play a crucial role in cell-to-cell communication and serve as carriers for a variety of biomolecules such as proteins, lipids, and nucleic acids. The most common method of classifying EVs is based on their biogenesis pathway, distinguishing exosomes, microvesicles, and apoptotic bodies as the major types. In recent years, there has been a growing interest in PDEs, as they offer a practical and eco-friendly alternative to exosomes sourced from mammals. Mounting data from both laboratory-based and animal model experiments indicate that PDEs have natural therapeutic properties that modulate biological activities within cells, demonstrating properties such as anti-inflammatory, antioxidant, and anticancer effects that may aid in treating diseases and enhancing human well-being. Moreover, PDEs hold promise as reliable and biologically compatible carriers for drug delivery. Although studies conducted before clinical trials have yielded encouraging results, numerous unresolved issues and gaps in understanding remain, which must be resolved to facilitate the effective advancement of PDEs toward medical use in human patients. A key concern is the absence of unified procedures for processing materials and for obtaining PDEs from different botanical sources. This article provides a comprehensive summary of existing findings on PDEs, critically examining the hurdles they face, and highlighting their substantial promise as a novel class of therapeutic tools for a range of illnesses.

## 1. Introduction

**Figure 1:**
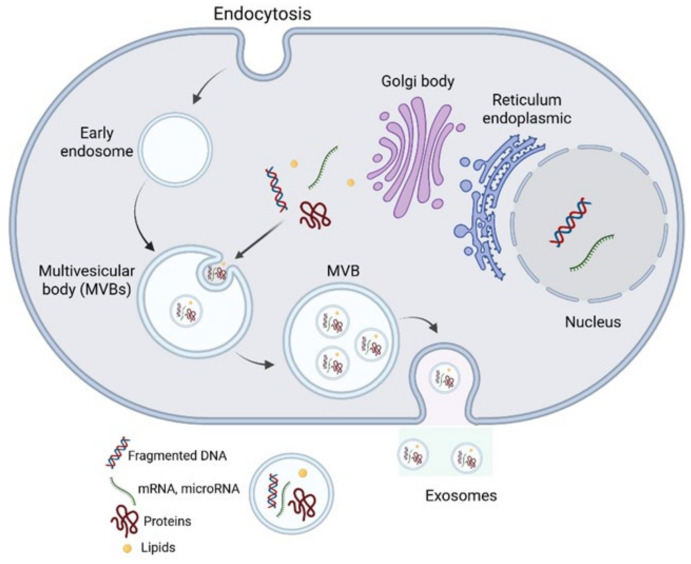
The biogenesis of EVs: the model for intracellular endocytic invagination and pinching of the plasma membrane to form endosomes and the inward budding of late endosomes, which take up cytosolic contents (proteins, nucleic acids, and metabolites), forms MVBs. As a result, MVBs may fuse with the plasma membrane at certain points to release the internal vesicles called “EVs”. Microvesicles, on the other hand, are formed because of the plasma membrane protruding or blebbing outward. In microvesicles, varieties of charges are packed into protrusions, which are pinched off the parent cell.

EVs are a heterogeneous group of membrane-bound vesicles that are believed to be produced and secreted by nearly all cell types under both physiological and pathological conditions [1]. EVs are crucial vehicles for intercellular communication over both short and long distances and can deliver a wide range of cargos including proteins, lipids, and various species of nucleic acids (long and small non-coding RNA, DNA, mRNA) effectively. Their ease of crossing biological membranes makes EVs interesting natural vectors for the delivery of drugs. Similarly, their presence in a variety of body fluids makes them a potential biomarker for early diagnosis, prognostication, and surveillance of cancer [2]. EVs play significant roles in multiple physiological processes including stem cell differentiation [3], autophagy [4], blood clotting [5], angiogenesis [6], immunity (innate and acquired) and immunomodulation [7], pregnancy [8], embryo implantation, reproduction, placental physiology, semen regulatory function [9], and tissue regeneration [10]. First, EVs are currently classified as exosomes, microvesicles, microparticles, ectosomes, oncosomes, and apoptotic bodies based on their size, origin, and characteristics. However, this classification is expanding to encompass the full heterogeneity of EVs, given the variation in cargo and their diverse roles. Exosomes are the best characterized and are less variable in size (ranging from 40 to 150 nm) compared to other subtypes. Exosomes are produced through membrane invaginations of endosomes, leading to the formation of multivesicular bodies (MVBs). These MVBs contain exosomes in the form of intraluminal vesicles (ILVs), which are released into the extracellular space when MVBs merge with the plasma membrane (Figure 1). These vesicles differ in their origin, size, molecular mechanisms, and cargo content.

In recent years, PDEs have attracted growing interest due to their distinctive ability to facilitate communication between different species and their positive impact on human health. Like mammalian vesicles, PDEs contain lipids, proteins, RNA, and other bioactive molecules. Their composition can vary depending on the plant species and the physiological conditions. They can transfer defensive molecules to target pathogens, helping the plant’s immune response. Additionally, they participate in signaling pathways that regulate plant growth and development and can influence the physiology of other organisms, including microbes and potentially animals [11]. PDEs are being explored for their potential in drug delivery systems, given their natural origin and biocompatibility. Their potential health benefits are being studied, particularly their antioxidant and anti-inflammatory properties [12].

Here, we discuss the potential of these natural nano-vesicles as cargoes and carriers of many important molecules implicated in physiological and pathological conditions.

However, many challenges remain in evaluating PDE-based therapeutics, but we are hopeful that current research will inspire further clues.

## 2. PDEs as a Cargo of Bioactive Compounds

**Figure 2:**
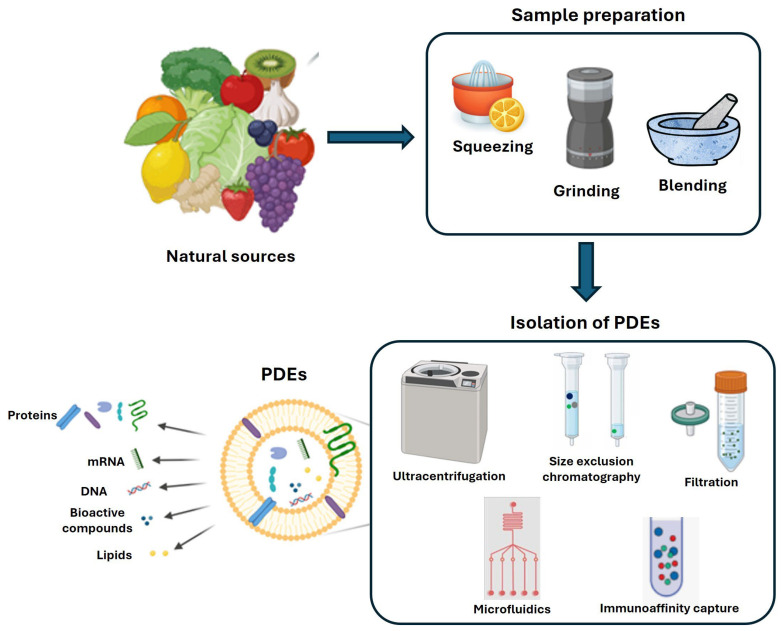
PDEs, extraction method from fruits and vegetables. Schematic representation of sample preparation and isolation methods of EVs. Description of main PDEs bioactive content. Created by BioRender scientific illustration software version 04.

EVs and exosomes are typically isolated from a conditioned cell culture medium [13] and extracellular fluids [14] (e.g., plasma/serum, urine, saliva, semen, breast milk, and cerebrospinal fluid). Exosomes contain many functional biomolecules, such as proteins, lipids, and nucleic acids (mRNA, microRNA, rRNA, and long non-coding RNA) [15]. Regardless of the exosome source, they share some characteristics, including a membrane composition that is rich in sphingomyelin, and ceramide. Additionally, exosomes contain various specific sets of proteins, including integrins, tetraspanin family proteins (CD9, CD63, and CD81), heat shock proteins (HSP60, HSP70, and HSP90), MVB synthesis proteins (Alix and Tsg101), membrane transporters, fusion proteins (such as Annexins, Rab GTPases, and flotillins), and cytoskeletal proteins. Thanks to online resources like ExoCarta, EVpedia, and Vesiclepedia, we now have insight into the content of EVs released by a wide range of species, from protozoa to humans. The composition of specific PDEs should mirror that of the cell of origin, influencing the biological activity of the vesicle. Indeed, there are numerous studies underway since there are still no specific markers (surface proteins or small RNA profiling) for plants or fruits. Soon, PDEs, like mammalian exosomes, could be considered as leading actors of intercellular communication, mediating both physiological and pathological responses. Traditional methods for isolating EVs, including exosomes, are primarily based on their size and buoyant density, including ultracentrifugation, filtration techniques, and gel filtration. Later, approaches that rely on changes in EV solubility and/or aggregation, such as precipitation methods, were developed. Recently, numerous advanced methods have been advanced, emphasizing precise interactions with surface molecules on EVs or utilizing microfluidic systems [16]. Among these, ultracentrifugation is widely considered the “gold standard” for EV isolation (Figure 2).

### 2.1. PDEs Protein Composition

PDEs obtained from a variety of plant species reveal a wide array of proteins belonging to multiple functional categories. Among them are proteins associated with responses to environmental challenges and pathogenic threats, regulation of metabolic pathways, modification of the cell wall structure, organization of the cytoskeleton, intracellular transport mechanisms, and components involved in secretion processes [17]. Liu et al. identified several plasma membrane proteins in garlic chive-derived EVs, including plasma membrane adenosine triphosphatase (ATPase), ATP-binding cassette transporter G family member (ABC transporter G family), pleiotropic drug resistance proteins, and hypersensitive induction response proteins [18]. Similarly, citrus-derived EVs are rich in membrane transporter proteins [19]. By comparison, Zhang et al. reported that ginger-derived EVs contain relatively low protein levels, mainly consisting of cytosolic proteins like actin and proteolytic enzymes, with only a small proportion of membrane proteins, such as membrane channels and transporters [20].

#### 2.1.1. Membrane Protein

Proteins found in PDEs are recognized as playing a crucial role in plant cellular activities. For example, membrane proteins could be involved in aiding the uptake and internalization of PDEs by mammalian cells. A study reported that the surface protein of PDEs derived from *Allium sativum* (II Lectin) may control their absorption by HEPG2. Subsequently, authors found a significant decrease in the absorption of PDEs after inhibiting CD98 receptors on HEPG2. In detail, Song et al. indicated that the interaction of lectin-like protein from PDEs with the glycoprotein CD98 on the cell surface facilitated the uptake of *A. sativum*-PDEs. This finding offered deeper insight into the mechanisms by which cells internalize PDEs [21]. In plants, the presence of aquaporins (AQPs), another type of membrane protein, has been associated with maintaining the integrity of the plasma membrane in PDEs and regulating their ability to transport water. AQPs have been identified in EVs derived from citrus fruits, grapes, and broccoli [22,23]. Patellins, which are transport-related proteins, may participate in intracellular membrane dynamics related to the formation of the cell plate during the final stage of cell division. These proteins bind to specific hydrophobic compounds and assist in transferring them across different parts of the cell. Therefore, having such proteins in PDEs is considered essential for supporting physiological functions in plants. Multiple Patellin isoforms (Patellin 1, 2, and 3) have been detected in EVs from Arabidopsis and citrus [24,25]. Furthermore, Syntaxins are regarded as central components in the processes of vesicle trafficking, membrane fusion, and exocytosis, playing crucial roles in several secretory functions. PDEs derived from Arabidopsis and citrus have also been shown to include Syntaxins [24]. Additionally, various RAS-related proteins—including RabA2a, RabA2b, RabB1c, Rab7, Rab2A, 5C, 6A, 7A, 8A, 11A, and 18—have been identified in EVs from Arabidopsis and citrus. These proteins are typically found in PDEs to support cargo selection, vesicle movement, and release [23].

#### 2.1.2. Cytosolic Protein

Recent research has demonstrated that the cytoplasmic proteins present in PDEs contribute to the defense against invading pathogens. For instance, Rutter et al. analyzed the proteomics of PDEs from Arabidopsis leaves and identified a high concentration of proteins linked to stress defense. Among these, they identified several proteins involved in signal transmission, many of which are highly induced in response to stress and contribute to immunity. Notably, one of these proteins is Rpm1-Interacting Protein4 (RIN4). RIN4 associates with disease resistance proteins, and when bacterial effectors modify RIN4, it triggers the activation of resistance protein signaling, leading to the initiation of an immune response. The presence of RIN4 and other proteins related to immune signaling indicates that EVs could be involved in immunity triggered by microbe-associated molecular patterns or effectors [26]. Stress-responsive proteins play a key role in helping plants withstand challenging environmental conditions. EVs originating from rice have been reported to include heat shock proteins (HSP) and chaperonins, which help prevent heat stress and support correct protein folding under stress conditions. HSPs (HSP60, 70, 80, and 90) have been identified in EVs derived from citrus fruits, sunflower, olive, grapes, and tomatoes [27]. In addition, the Annexin family, in particular Annexin A1 and Annexin A2, are critical in EV biogenesis, in the budding of the ILVs, and in the multivesicular bodies generation. Annexins were found in EVs from citrus species, sunflower seedlings, and EV isolates from the leaf apoplast [28]. Despite numerous studies on the protein composition of PDEs, a standardized and specific marker protein for PDEs, along with a comprehensive protein library, has yet to be established. Conducting large-scale proteomic analyses on EVs from various plant sources and classifying them based on the findings could significantly advance the in-depth research of PDEs.

### 2.2. PDEs Lipid Composition

Lipids are essential elements of the lipid bilayer in PDEs, and their composition differs significantly from that of vesicles derived from mammalian cells and synthetically produced liposomes. The lipid composition of the vesicle membrane plays a crucial role in facilitating intercellular interactions and ensuring vesicle stability under both physiological and pathological conditions [29]. PDEs lipid profiling has determined that two major classes of lipids are found such as phospholipids and glycerol lipids, but they lack cholesterol. The primary types of phospholipids found in PDEs comprise phosphatidic acid (PA), phosphatidylethanolamine (PE), phosphatidylcholine (PC), and phosphatidylinositol (PI), while the two predominant glycerol lipid groups are digalactosyldiacylglycerol (DGDG) and monogalactosyldiacylglycerol (MGDG) [30,31]. Lipid profiles can differ among PDEs obtained from various plant origins. As an example, PDEs extracted from orange juice were reported to contain PE (40%), PC (25%), PI (12%), and PA (5%) [32]. In addition, independent lipidomic investigations by two research groups have consistently identified PA—comprising more than 30% of total lipids—and DGDG as key constituents of ginger-derived nanovesicles [20,33]. In a similar manner, PDEs from turmeric rhizomes were found to have a high concentration of DGDG (41.6%) and PA (19.7%) [34]. Lipids possess numerous biological functions; for example, PA is known to activate intracellular signaling pathways such as mTOR, a serine/threonine-protein kinase involved in regulating processes like cell growth, proliferation, and survival. PA may also initiate mitogen-activated protein kinase (MAPK) pathways, potentially accounting for the influence of PDEs on cellular development and expansion [35]. One study demonstrated that nano-vesicles derived from orange juice (ODVs) are enriched in PA, which regulates their action on the mTOR pathway. This could help explain the impact of ODVs on villi growth in mice fed a high-fat, high-sugar (HFHS) diet. Furthermore, the study showed that ODVs could reverse diet-induced gut alterations in obese mice [32]. The rapid incorporation of ODVs into intestinal barriers (IBs) in vitro supports previous research showing that fruit-derived nanovesicles can be captured by intestinal cells (e.g., intestinal dendritic cells for broccoli nanovesicles, macrophages for grapefruit nanovesicles, and Lgr5+ stem cells for grape nanovesicles) and similarly could induce cell growth (i.e., in vitro on Caco-2 cells and in vivo in jejunum. Additionally, it was demonstrated that PC, sn-glycero-3-phosphocholine (POPC), and, to a lesser extent, PE contained in ODVs are essential components of mucus and possess anti-inflammatory properties. For example, patients with ulcerative colitis showed significant restoration of the mucosal barrier following treatment with PC. Therefore, the improvement in intestinal function induced by ODVs in HFHSD mouse jejunum may be linked to the PC and POPC present in ODVs [32]. Additionally, PA is notable among phospholipids for its role in the fusion and fission processes of biological membranes, contributing to membrane dynamics. In addition, PA is involved in vesicular trafficking, secretion, and endocytosis, likely through its effects on cytoskeletal organization [36]. Furthermore, the role of EVs and their cellular uptake ability are increasingly associated with their lipid makeup and the organization of their lipid structures. In particular, ginger-derived PDEs rich in PA were predominantly internalized by *Lactobacillus rhamnosus*, whereas those from grapefruit containing higher levels of PC were more efficiently assimilated by members of the intestinal Ruminococcaceae family. These findings imply that distinct lipid types and their specific arrangements may serve as recognition cues for selective absorption by various recipient cells, thereby offering the possibility of cell-specific therapeutic effects, as supported by previous research [31]. Sundaram et al. examined the impact of ginger-derived vesicles on the periodontal pathogen *Porphyromonas gingivalis*, emphasizing the crucial role of phosphatidic acid (PA) in facilitating bacterial uptake of extracellular vesicles. Their findings showed that the proportion of PA significantly affects the binding of EVs to hemin-binding protein 35, thereby modulating the virulence of *P. gingivalis* [37]. The identification of specific lipids has been recognized as a key factor in directing EVs to target tissues. For example, PC appears to promote the movement of EVs from the intestine to the liver, whereas PA may enhance their retention and accumulation within the intestinal tract [38]. Conversely, the lipid constituents of PDEs also demonstrate protective and regenerative effects on tissues. For instance, the PA present in ginger-derived exosomes can induce the phosphorylation and expression of fork-head box protein A2b (Foxa2) in intestinal epithelial cells, thereby influencing the secretion of exosomes to prevent insulin resistance [39]. Research has demonstrated that PDEs from grapes, sunflowers, and ginger also contain PA. In fact, Wang et al. [40] reported that PC and PE are the dominant lipids in vesicles derived from grapefruit, contributing to their strong antioxidant, anti-inflammatory, and anti-colitic effects. Among other lipid molecules, PDEs frequently contain DGDG and MGDG—key glycolipids that play vital roles in various cellular processes [40]. It has been shown that MGDG and DGDG have been linked to the anti-inflammatory and cancer benefits of a green leafy vegetable diet in humans due to their ability to regulate the levels of free radicals like nitric oxide [41]. In another study, it has been reported that MGDG has an anti-inflammatory activity in human articular cartilage and possibly activates an anti-inflammatory loop triggered by COX-2, indicating a potential role of COX-2 in resolution of inflammation [42].

### 2.3. PDEs microRNAs Composition

MiRNAs are small non-coding RNAs that play a crucial role in regulating gene expression at the post-transcriptional level by targeting specific mRNAs for degradation or inhibiting their translation. Over the past decade, the potential of plant-derived miRNAs as functional food components with therapeutic benefits, such as enhancing antitumor immunity and preventing diseases, has been proposed [43]. Traditionally, the health benefits of plants were attributed to compounds like polyphenols, alkaloids, saponins, tannins, vitamins, and minerals. However, recent research has shown that plant miRNAs can be absorbed by the mammalian digestive system and influence gene expression and cellular functions in other species, revealing cross-species regulatory interactions [28]. Although experimental findings remain inconclusive, plant miRNAs are suggested to exert biological effects within recipient cells. For example, rice-derived miR-168a, commonly detected in the serum of individuals in China, has been shown to downregulate low-density lipoprotein receptor adaptor protein 1 in the livers of mice, resulting in reduced plasma clearance of LDL [44]. More recently, oral delivery of plant-derived miR-2911 was found to attenuate liver fibrosis in murine models [45]. Likewise, miR-156a sourced from cabbage, spinach, and lettuce has been linked to protective effects against atherosclerosis [46]. Another investigation revealed that abundantly expressed miRNAs in PDEs isolated from 11 commonly consumed plants could modulate the expression of genes involved in inflammation and cancer in vitro, thereby indicating a potential role in cross-species cell-to-cell signaling. For instance, miR-168c is believed to influence TSC22D3 gene expression, which encodes a glucocorticoid-responsive protein with immunosuppressive and anti-inflammatory properties. As a result, EVs may modulate glucocorticoid function by affecting this gene, suggesting possible immunomodulatory capabilities. Furthermore, miR-156c and miR-159a—known regulators of the TNF-α signaling pathway in adipocytes and inflammatory processes—have been identified in walnut-derived exosome-like nanoparticles [47]. In another research, Peng and colleagues discovered that co-culturing bone marrow mesenchymal stem cells (BMSCs) with ginseng-derived nanovesicles led to the uptake of various miRNAs from the PDEs into the BMSCs. These miRNAs were found to target genes involved in regulating neural differentiation. Moreover, the researchers noted a significant enhancement in neural differentiation in BMSCs exposed to the ginseng-derived nanovesicles [48]. Nanovesicles derived from blueberries, containing miR-156a, miR-162, and miR-319d, demonstrated potential in protecting the human endothelial cell line (EA.hy926) against the harmful effects of tumor necrosis factor-α (TNF-α) [49]. Therefore, miRNAs derived from PDEs hold significant therapeutic potential. Given their ability to facilitate intercellular communication, further research on PDEs could pave the way for novel therapeutic strategies in the future [50].

### 2.4. Natural Bioactive Compounds

Plants have been well known for producing essential primary and secondary bioactive compounds involved in metabolic processes. Numerous studies have indicated a possible function of PDEs in plant defense mechanisms and intercellular communication, suggesting that PDEs carry a wide variety of plant metabolites [51]. For example, PDEs found in orange juice have been identified to include primary metabolites such as carbohydrates (glucose, fructose, and sucrose) and amino acids (alanine, asparagine, isoleucine, threonine, and leucine). Likewise, vesicles derived from grapefruit have been reported to contain carbohydrates, amino acids (leucine and isoleucine), alpha hydroxy acids (glycolic and citric acids), and fatty acids (palmitic acid and doconexent) [32]. However, it has been observed that not all secondary metabolites produced by plants are included in PDEs. For example, although orange juice contains vitamin C and naringenin, these bioactive compounds were absent in orange-derived PDEs. This indicates that it remains unclear how the components of PDEs become encapsulated within PDEs throughout their formation [32]. Gingerols and shogaols are well-known for their beneficial effects on human health and nutrition. Both compounds display a variety of biological activities, including anticancer, antioxidant, antimicrobial, anti-inflammatory, anti-allergic, and several effects on the central nervous system. One study confirmed the presence of the bioactive compounds 6-gingerol and 6-shogaol inside ginger-derived PDEs, and therefore, the anti-inflammatory effects of these PDEs can be linked to both 6-gingerol and 6-shogaol [52]. Wang et al. also revealed that naringin, a primary flavonoid in grapefruits, along with its active form naringenin, was found in grapefruit-derived PDEs [53]. Considering the extensive pharmacological properties of naringenin, the high presence of these bioactive metabolites in grapefruit-derived PDEs likely contribute to their pharmacological effects [54]. Interestingly, in a separate study, substances with recognized anti-cancer potential, such as myo-inositol, quininic acid, aucubin, and doconexent, have likewise been found in grapefruit vesicles [55]. Tea leaf-derived nanovesicles contain a variety of biologically active compounds, including gallic acid, caffeine, epigallocatechin gallate (EGCG), and flavones like quercetin. These constituents are well known for their antioxidant, anti-inflammatory, and anti-cancer properties, suggesting that nanovesicles derived from tea leaves may have potential medical uses [56]. Likewise, Aloe vera-derived vesicles contained high levels of active constituents such as aloe-emodin, a potent anti-cancer agent, along with aloesin and β-sitosterol, both of which are acknowledged for their antioxidant activity [57]. Strawberry-derived PDEs are particularly abundant in vitamin C [58]. Apple-derived EVs include flavonoids and furanocoumarins, which have been shown to exhibit toxicity toward certain types of fungi [59]. PDEs from Javanese ginger and turmeric carry curcuminoids, recognized for their antioxidant properties [60]. Tobacco-derived PDEs contain alkaloids and phenolics, whereas EVs from aconiti tuber are rich in trace amounts of aconitine, hypaconitine, and mesoaconitine, all of which are toxic in nature [30]. Thus, PDEs are filled with a diverse range of metabolites depending on their source, indicating significant potential for future therapeutic applications.

## 3. Therapeutic Activities of PDEs

**Figure 3:**
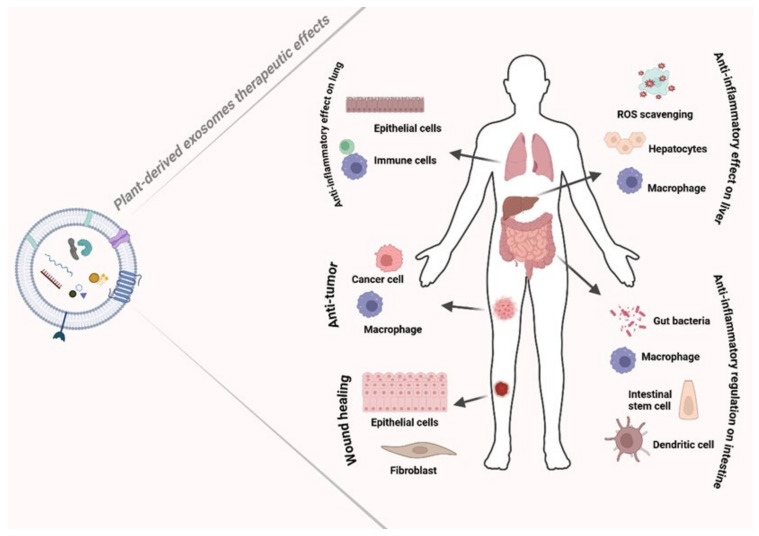
Representative scheme of the therapeutic effects associated with EVs like exosomes, as wound healing, anti-tumor, and anti-inflammatory effects. Created by BioRender scientific illustration software.

PDEs therapeutic activities include promoting wound healing, exhibiting anti-inflammatory effects, modulating innate immune cells, exhibiting antioxidant activity and anti-cancer properties, delivering hepatoprotective benefits, and preventing apoptosis [35] (Figure 3).

### 3.1. Anticancer Activity of PDEs

Tumors and malignancies have always been a significant challenge to modern medicine. Traditional cancer therapies, such as chemotherapy and radiotherapy, work by either inhibiting cell proliferation or inducing apoptosis to eliminate tumor cells. However, these approaches often face high rates of treatment failure and severe side effects due to drug resistance and lack of selective targeting. The limitations of current treatments highlight the urgent need for new and innovative cancer treatment options. Numerous investigations have shown that PDEs exhibit anti-proliferative or pro-apoptotic effects, which play a role in supporting their anti-cancer activity. Consequently, there is increased interest in using PDEs as anticancer therapeutics [61]. For instance, vesicles derived from tea flowers (*Camellia sinensis*) (TFDVs) have demonstrated significant cytotoxicity against breast cancer cells. This effect leads to mitochondrial damage and cell-cycle arrest due to the increased production of reactive oxygen species (ROS). Elevated ROS levels can not only cause mitochondrial damage but also disrupt the cell cycle, resulting in reduced cell proliferation, migration, and invasion in breast cancer cells. Further research using xenograft mouse models showed that mice either treated with these exosomes, intravenously or orally, had an increased survival rate. Additionally, TFDVs administered via intravenous injection or oral intake were found to accumulate in breast tumors and lung metastases, effectively inhibiting tumor growth and metastasis. This study offers valuable insights into using natural exosomes to combat breast cancer and its metastases through both intravenous and oral delivery methods [62]. Another study revealed that nano-vesicles isolated from *Citrus sinensis*, *Citrus limon*, *Citrus paradisi*, and *Citrus aurantium* effectively reduced the proliferation of cancer cells in breast, skin, and lung tissues, while exhibiting minimal toxicity toward healthy human keratinocytes (HaCaT). Grapefruit-derived nanovesicles (GFDVs) were shown to reduce cell viability by approximately 40% in various tumor cell lines. Analysis of melanoma cell lines revealed that GFDVs cause cell cycle arrest at the G2/M phase, which is linked to decreased levels of cyclins B1 and B2 and an increase in the cell cycle inhibitor p21. The reduction in cyclin B2 hinders invasiveness and prevents metastasis. Further evidence suggests that GFDVs inhibit cancer cell viability by blocking Akt and ERK signaling pathways. Additionally, GFDVs exhibit pro-apoptotic effects through the activation of PARP-1, thereby limiting cancer cell proliferation. The metabolomic profile of GFDVs indicated high levels of alpha hydroxy acids, leucine/isoleucine, myoinositol, and doconexent, compounds known for their anti-proliferative properties. This bioactive metabolomic content is believed to underline the well-documented anti-tumor effects of GFDVs [55]. Another team of researchers showed that vesicles isolated from Citrus lemon (CLDVs) affected both cell death-promoting and survival-promoting pathways when applied to lung, colon, and leukemia cancer cells. This led to increased mRNA expression of pro-apoptotic molecules such as Bad and Bax, while reducing the levels of pro-survival molecules like Survivin and Bcl-xl. TRAIL (TNF-related apoptosis-inducing ligand), which plays a role in immune defense against viruses and cancer cell surveillance, along with its receptor Dr5, were also found to be upregulated at the mRNA level in cells treated with CLDVs. To confirm the pro-apoptotic effects of these nanovesicles, the researchers used an in vivo xenograft model for chronic myeloid leukemia (CML) and observed that CLDV administration significantly reduced tumor growth. Additionally, the in vivo results confirmed that this tumor-suppressing effect was not only due to TRAIL-induced apoptosis but also involved the inhibition of angiogenesis, as shown by decreased levels of pro-angiogenic cytokines, including VEGF-A, IL6, and IL8 [63]. A proteomic analysis performed by the same team of researchers revealed that the cancer-suppressing effect of CLDVs, particularly against colorectal tumors, was associated with the downregulation of Acetyl-CoA carboxylase 1 (ACACA). ACACA serves as a key metabolic regulator for proteins, lipids, and carbohydrates, and is heavily involved in reprogramming various metabolic pathways in cancer cells [64]. A different study on vesicles derived from lemon (LDVs) in various gastric cancer cell lines (AGS, BGC-823, and SGC-7901) revealed that these vesicles induced cell cycle arrest and triggered apoptosis. The study elucidated that LDVs anti-tumor mechanism operates through the generation of ROS, which in turn upregulates GADD45A. GADD45A is known to play a crucial role in cellular responses to both internal and external stressors, including DNA repair and control over the cell cycle. Its overexpression usually results in S-phase cell cycle arrest and suppression of cell proliferation. This cascade ultimately results in the S-phase cell cycle arrest of gastric cancer cells and initiates apoptosis. Furthermore, lemon-derived vesicles were shown to be safe, as no notable pathological changes were observed in key organs (heart, liver, spleen, lung, and kidney) following administration in mouse models [65]. Additionally, nanovesicles from Citrus lemon were reported to suppress the proliferation of colorectal cancer cells lacking functional p53, through the macropinocytosis pathway. The study’s findings indicate that p53 loss increases macropinocytic activity, and the nanovesicles exert their anti-proliferative action via this mechanism [66]. One investigation explored the effects of ginger rhizome-derived vesicles (GDVs) on triple-negative breast cancer (TNBC), an aggressive form of breast cancer. GDVs caused a dose-dependent reduction in the viability of MDA-MB-231 breast cancer cells, while leaving normal cell lines unaffected. GDVs induced apoptosis is indicated by morphological changes, nuclear fragmentation, membrane damage, phosphatidic serine translocation, ROS generation, drop in mitochondrial membrane potential, expression of apoptotic specific proteins, and increased caspase activity. GDVs also induced cell cycle arrest, inhibited cell migration, and suppressed colony formation in TNBC cells [67]. In line with this study, another research group recently demonstrated that ginger-derived nanovesicles have an anti-cancerous effect on breast cancer cells (MCF7). Rhizome of ginger is rich in phenolic and flavonoids contributing to antioxidant and anticancer activity. Their analyses have shown that a significant increase in caspase-3, −8, and −9 mRNA levels was observed in ginger extract treated MCF-7 cells compared to untreated control cells. Furthermore, their data also showed that ginger extract significantly reduces the mRNA levels of anti-apoptotic genes Bcl-2 and Bcl-xL while increasing the levels of apoptosis-promoting genes (Bax, p53, and p21) in cancerous cells [67]. Moreover, garlic-derived vesicles significantly inhibited the proliferation of kidney and lung cancer cells (A498 and A549) by inducing S-phase cell cycle arrest and caspase-mediated apoptosis [68]. Moreover, garlic-derived vesicles markedly suppressed the growth of renal and pulmonary cancer cells (A498 and A549) through the induction of S-phase arrest and caspase-dependent apoptosis [68]. Beyond triggering apoptosis and halting the cell cycle, PDEs also seem to influence the tumor microenvironment. Vesicles derived from ginseng were reported to reprogram macrophage polarization both in vitro and in vivo via a TLR4-MyD88 signaling pathway, ultimately leading to tumor suppression. Additionally, these vesicles demonstrated excellent biocompatibility, showing no harmful effects on normal cells or in murine models [69]. Another important aspect is that PDEs may utilize different mechanisms across various tumor types. In vitro studies on vesicles derived from bitter melon showed reduced cell viability and proliferation, along with the suppression of glioma cell invasion and motility in U251 cells. Nevertheless, flow cytometry results did not support apoptosis induction. Further analysis confirmed that the vesicles’ anticancer potential was mediated through inhibition of glioma growth via the PI3K/AKT pathway and suppression of cancer spread through decreased expression of matrix metalloproteinase 9 (MMP9) [70]. Emerging research indicates that the antitumor effects of PDEs may be driven by multiple underlying mechanisms. These include (1) apoptosis specifically targeting cancer cells (through TRAIL- and caspase-mediated pathways), potentially involving the regulation of ACACA, GADD45a, Bcl-2, and mitochondrial membrane potential; (2) induction of cell cycle arrest at either the S phase or G2/M checkpoint; and (3) reprogramming of the tumor microenvironment, particularly via macrophage polarization regulated through the TLR4-MyD88 signaling axis [51].

### 3.2. Anti-Inflammatory Activity of PDEs

In recent years, several reports show that PDEs can counteract inflammatory symptoms, and research on PDEs has been accelerated to elucidate the emerging application of this naturally derived nanoparticle in anti-inflammatory therapy [71]. Inflammation is beneficial for protecting the body, but excessive inflammation is seen because of an imbalance in immune responses and can negatively impact on the treatment mechanisms and progression of various diseases, such as inflammatory bowel diseases. Through modification and transformation, PDEs can target the inflammatory microenvironment, inhibit the harmful immune response in the inflammatory tissues, and promote the survival and regeneration of damaged parenchymal cells. The possible biological pathways involved in the effects of PDEs were explored, emphasizing their role in modulating the immune system, reducing oxidative stress, preventing apoptosis and necrosis in damaged cells, and supporting their survival and repair [72].

#### 3.2.1. Immune Regulation

Many studies have shown that PDEs have great potential in regulating immune function and inflammatory response in in vitro and in vivo models. PDEs can deliver autoimmune regulatory cargoes (such as RNA, protein) to immune cells (M1 macrophages, DC cells, CD4 + Th1, and Th17 cells) and phenotypically convert them into immunosuppressive cells (M2 macrophages, tolerant DC cells, and regulatory T cells) [72]. Macrophages are essential mediators of the immune system, playing key roles in tissue-specific functions, maintaining tissue homeostasis, and initiating inflammatory responses. M1 macrophages are associated with pro-inflammatory activity, while M2 macrophages exhibit anti-inflammatory activity. Recent studies suggest that macrophage polarization from M1 to M2 phenotype might be exploited to prevent tissue injury caused by inflammation [73]. Through vitro experiments, Wu et al. demonstrated that EVs derived from Pueraria lobata, an edible and medicinal herb, were taken up by isolated mouse macrophages and shifted M1 macrophages toward M2 by transferring functional cargoes [73]. One study showed that ginger-derived vesicles (GDVs) induce the proliferation of M2 macrophages. They demonstrated that GDVs inhibit NF-kB activation in inflammatory conditions, thereby exerting anti-inflammatory effects, as NF-kB activation is significantly heightened in inflammation-related diseases. Thus, it can be inferred that the sequential signaling interactions play a role in M2 macrophage proliferation. They also observed that GDVs upregulate the IL-10, anti-inflammatory cytokine, in mice and downregulate pro-inflammatory cytokines [74]. However, the detailed molecular mechanism regulating the function of macrophages in these studies remains to be determined [72]. Bian et al. found that garlic-derived EVs targeted delivery of miRNA-396e of macrophages to reduce the expression of fructose-2,6-diphosphatase 3 (PFKFB3), one of the critical glycolytic enzymes, thereby transforming LPS-induced metabolic reprogramming of M1 macrophages into M2 [75]. Another research showed that tomato fruit-derived EVs were able to reduce the miRNA levels of pro-inflammatory factors IL-1β and IL-6 in LPS-stimulated human monocytes [76]. A study on apple-derived vesicles (ADVs) demonstrated their anti-inflammatory potential. When type 1 macrophages were treated with ADVs, there was a notable reduction in the expression of IL-1β and IL-8, two key pro-inflammatory cytokines. Further analysis indicated that this anti-inflammatory effect is primarily driven by the induction of miR-146 production in M2 macrophages. The findings revealed that increased levels of miR-146a, along with suppressed IL-1β expression, point to inhibition of the NF-κB signaling pathway in activated macrophages following ADV treatment. Numerous in vitro and in vivo studies have consistently shown that miR-146a plays a pivotal role in downregulating pro-inflammatory cytokine production by targeting and suppressing the NF-κB pathway [77]. Furthermore, a separate investigation conducted by Teng et al. revealed that ginger-derived PDEs effectively suppressed the expression of SARS-CoV-2 S and viral Nsp12 genes in vitro, and helped alleviate lung inflammation in vivo, an effect attributed to the miRNA content of the ginger-derived vesicles [78].

#### 3.2.2. Inflammatory Bowel Disease

Inflammatory bowel disease (IBD), which includes Crohn’s disease and ulcerative colitis, is a chronic autoimmune condition marked by persistent inflammation. In recent years, a growing body of evidence has indicated that PDEs hold promise as an emerging therapy for IBD and may partially overcome the drawbacks of conventional treatments. PDEs possess distinctive structural and functional characteristics that render them particularly well-suited for IBD management. Their lipid bilayer membranes, as an example, are vital for enhancing stability against enzymatic degradation in the gastrointestinal environment and support their inherent anti-inflammatory activity. Moreover, PDEs can positively influence intestinal healing processes and regulate immune activity through multiple pathways, helping to restore the delicate equilibrium between inflammatory and anti-inflammatory mediators, thereby mitigating IBD progression. Exosomes purified from plant extracts like pineapple, grapefruit, and grape—carrying miRNAs—have been demonstrated to be taken up by intestinal cells within the mammalian small intestine [79]. This represents a promising strategy for targeting PDEs in the treatment of intestinal inflammation. In DSS-induced colitis models, grape-derived vesicles (GRDVs) were shown to cross the intestinal mucus layer and stimulate the Wnt/β-catenin pathway, leading to enhanced proliferation of Lgr5+ intestinal stem cells. This, in turn, facilitated mucosal tissue repair and promoted swift restoration of overall intestinal function [80]. Another study showed that PDEs from turmeric (TDVs) could reduce the expression of inflammatory cytokines and promote the transformation of M1 to M2. Importantly, TDVs alleviated the colitis-related symptoms through restoring the intestinal epithelium barrier, regulating the composition and relative abundance of gut microbiota and reshaping the immune microenvironment [81]. Ginger, a plant valued for both its culinary and medicinal properties, produces exosome-like vesicles that have demonstrated multiple therapeutic effects in the context of inflammatory disorders. Earlier research showed that when ginger-derived vesicles were administered orally, they selectively enhanced the production of anti-inflammatory cytokines—IL-10 and HO-1—by triggering Nrf2 activation in RAW264.7 macrophages and stimulating the Wnt/TCF4 signaling pathway in the intestines of mice [82]. A separate group explored the impact of onion-derived vesicles on RAW264.7 macrophages. Onion extract is recognized for its potent anti-inflammatory properties, and the exosomes obtained from this extract exhibited similar effects. Moreover, these EVs significantly reduce inflammation by modulating the NF-κB signaling pathway. They also inhibit, in a dose-dependent manner, the expression of pro-inflammatory mediators such as IL-6, IL-1β, COX-2, and iNOS in RAW264.7 cells [83]. Intestinal immune balance can be influenced by broccoli-derived vesicles (BDVs), as demonstrated in three distinct mouse colitis models. AMP-activated protein kinase (AMPK) is a key regulatory enzyme and signaling component essential for maintaining immune system equilibrium. It is widely expressed in various immune cells, including colonic macrophages and dendritic cells (DCs), where it contributes to controlling cytokine production, apoptosis, and cellular proliferation—factors implicated in the pathogenesis of IBD. Research indicates that BDVs suppress the production of inflammatory cytokines like TNF-α, IL-17A, and IFN-γ in experimental colitis models. Furthermore, BDVs help block dendritic cell activation and promote the development of tolerogenic DCs via AMPK activation in colitic mice. Thus, BDVs play a vital role in preserving gut immune homeostasis and safeguarding against colitis in mice [84]. The NLRP3 inflammasome plays a central role in regulating innate immune responses, and its dysregulated activation has been linked to the development of several conditions, including inflammatory bowel disease and various auto-inflammatory disorders. Garlic chive-derived vesicles (GCDVs) have demonstrated a dose-dependent ability to suppress key downstream events following NLRP3 inflammasome activation, such as caspase-1 autocleavage, inflammatory cytokine secretion, and pyroptosis in primary macrophages. This effect is primarily attributed to the presence of 1,2-dilinoleoyl-sn-glycero-3-phosphocholine, a bioactive lipid found in GCDVs [18]. In a similar manner, ginger-derived vesicles (GDVs) block the assembly of the NLRP3 inflammasome, reduce IL-1β and IL-18 production, and prevent pyroptotic cell death in primary macrophages, predominantly through their lipid constituents. In DSS-induced colitis models, orally delivered GDVs are efficiently internalized by intestinal epithelial cells (IECs) and macrophages, resulting in enhanced survival and proliferation of IECs. This uptake also downregulates the production of pro-inflammatory cytokines (TNF-α, IL-6, and IL-1β) while upregulating anti-inflammatory cytokines (IL-10 and IL-22). Consequently, GDVs hold great potential as therapeutic agents for alleviating colitis and promoting mucosal healing [85]. A recent study has demonstrated that vesicles from the edible barks of mulberry (MBDVs) promote the activation of the aryl hydrocarbon receptor (AHR) signaling pathway, an important key in modulating immune and inflammatory responses, which in turn leads to the induction of COPS8. Induced COPS8 is crucial for the prevention of DSS-induced mouse colitis by MBDVs through the induction of a variety of AMPs. AMPs are key components of the immune defenses in multicellular organisms and are found throughout both the animal and plant kingdoms [86]. Furthermore, a study found that vesicles derived from blueberries (BLDVs) can influence the TNF-α response. BLDVs counteracted the TNF-α-induced increase in mRNA levels of IL-6, IL1RL1, MAPK1, ICAM1, TLR8, and TNF, while also enhancing the antioxidant capacity of cells by upregulating HMOX1 and NRF1 in the human endothelial cell line EA.hy926 [49]. Apple-derived exosomes (ADVs) were also found to be internalized by human epithelial colorectal adenocarcinoma (Caco-2) cells in vivo after co-incubation. They discovered that the mRNA expression levels of various transporters, such as organic-anion-transporting polypeptide (OATP) 2B1, were altered in Caco-2 cells treated with ADVs. Furthermore, they found that the 3′-untranslated region of the OATP2B1 gene was required for the response to ADVs, suggesting that microRNA in the ADVs might be involved. These results suggest a novel mechanism in which large molecules, such as microRNA in food, may influence intestinal transporters through food-derived vesicles. This also emphasizes the promise of food-derived vesicles as vehicles for transporting biologically active macromolecules to intestinal tissues [59].

#### 3.2.3. Liver Diseases

An interesting study reported the hepatoprotective effect of ginger-derived vesicles (GDVs) against alcohol-induced liver damage in mice. The researchers observed that GDV-mediated activation of Nrf2 triggered the upregulation of several key genes involved in liver detoxification and antioxidant defense, including HO-1, NQO1, GCLM, and GCLC. Moreover, GDVs suppressed the generation of reactive oxygen species (ROS), which partially contributed to their liver-protective effects [33]. In another study, garlic-derived nanovesicles (GADVs) were isolated, and their effects on HepG2 cells were assessed in vitro. The findings showed that GADVs exerted anti-inflammatory activity by suppressing the expression of pro-inflammatory mediators in HepG2 cells [87]. Acute liver failure (ALF) is a critical condition that can be fatal, often resulting from viral infections or the misuse of drugs. In an investigation, the effect of grape-derived vesicles (GRDVs) was investigated in D-GalN/LPS acute liver failure mice. The results showed that treatment with GRDVs improved liver pathology and reduced the levels of soluble inflammatory mediators IL-6, IL-1β, and TNF-α in the serum of ALF mice. GRDVs reversed the upregulation of Cleaved Caspase-9, Cleaved Caspase-3, p53, and Bax expression and decreased Bcl2 activation caused by D-GalN/LPS, and inhibited NF-κB p65 expression and translocation to the nucleus. Also, treatment with GRDVs resulted in a significant reduction in NLRP3 activation and Caspase-1 maturation, as well as a decrease in the release of the inflammatory mediator IL-18. Furthermore, the detection of chemokines in mouse liver tissue revealed that GRDVs exhibited minimal reduction in the expression of CCL2, CCL3, CCL5, and CCL8. The decreased expression of CCR2 and CCR5 in the liver suggests that GRDVs could decrease the recruitment of monocytes from circulation to the liver. This study provides novel evidence that GRDVs can ameliorate inflammatory eruptions and hinder the migration of circulating monocytes to the liver, as well as decrease macrophage infiltration by inhibiting CCR2/CCR5 signaling [88].

#### 3.2.4. PDEs as Natural ROS Scavengers

Reactive oxygen species (ROS) are normally generated within cells as a byproduct of aerobic respiration but can also arise in response to exposure to foreign substances, inflammatory cytokines, or bacterial infections. ROS function like a double-edged sword: they are crucial for cell survival and proliferation, but an imbalance between ROS generation and the cellular antioxidant defense can convert ROS from being beneficial to harmful [89]. Excessive ROS generation gives rise to oxidative stress, which leads to molecular damage and contributes to the development of various disorders, including neurodegenerative conditions and cardiovascular diseases. Antioxidant therapies help counteract oxidative stress by neutralizing ROS and reducing the associated cellular damage. A range of small-molecule candidates, such as mimetics of antioxidant enzymes and Nrf2 activators, have been evaluated for their antioxidant potential. Despite encouraging outcomes in preclinical research, their performance in clinical trials has been relatively modest [90]. A recent investigation demonstrated that vesicles derived from strawberries (SDVs) possess antioxidant properties in vitro, offering protection to adipose-derived mesenchymal stem cells (ADMSCs) under oxidative stress conditions, with no notable toxicity observed. Notably, SDVs enhanced the viability of ADMSCs exposed to oxidative stress in a dose-dependent fashion and reduced intracellular ROS accumulation. Additionally, the presence of vitamin C—an established antioxidant—within these vesicles may play a role in their protective effect [58]. Exosomes extracted from the juice of Citrus lemon (CLDV) are taken up by human mesenchymal stromal cells (MSC) with ease and do not affect their survival. Upon exposure to H_2_O_2_, pretreated cells showed that CLDVs conferred dose-dependent protection to MSCs against H_2_O_2_-induced cytotoxicity, along with a significant reduction in ROS generation [91,92]. The antioxidant effects of PDEs via the nuclear translocation of Nrf-2 have also been reported. For instance, Kim and Rhee have shown that vesicles derived from carrots (CDVs) possess antioxidant properties. These CDVs were effective in shielding cardiomyoblasts (H9C2) and human neuroblastoma (SH-SY5Y) cells from oxidative stress. To assess the antioxidant activity of CDVs in H9C2 cells, oxidative stress was induced using H_2_O_2_. The results showed that CDVs markedly reduced both ROS production and apoptosis. In addition, treatment with CDVs led to an upregulation in the protein and mRNA expression of key antioxidant-related genes, including Nrf2, HO-1, and NQO1 [93]. An independent study also confirmed that carrot-derived PDEs enhanced the movement of Nrf2 into the nucleus [82]. Similarly, Xin et al. investigated the effect of grape-derived vesicles (GRDVs) in mice with GalN-induced acute liver failure. Interestingly, they found that GRDVs have the same effect as garlic-derived vesicles in mice with GalN-induced acute liver failure [88]. Fulminant hepatic failure (FHF) is a rare and severe liver condition with a poor prognosis. Recent studies have demonstrated that inhibiting the NLRP3 inflammasome can mitigate liver damage in mouse models. This research revealed that shiitake mushroom-derived vesicles (SDVs) effectively inhibited the activation of the NLRP3 inflammasome by preventing its assembly in primary macrophages. Furthermore, SDVs decreased the secretion of interleukin 6 (IL-6) and reduced both the protein and mRNA expression levels of the IL-1β gene. Remarkably, pre-treatment with SDVs protected mice from GalN/LPS-induced acute liver injury. Thus, SDVs, recognized as a powerful new inhibitor of the NLRP3 inflammasome, emerge as a promising group of agents with potential to treat FHF [88,94].

#### 3.2.5. PDEs Regenerative Activity

**Figure 4:**
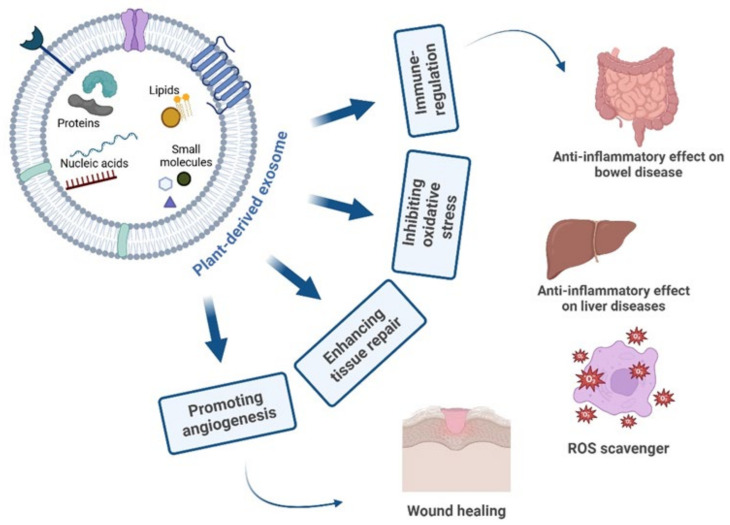
Plant-derived exosomes property. Cargo of PDEs promote angiogenesis, tissue repair, and immune regulation, and also present anti-oxidative activity. Created by BioRender scientific illustration software.

Given the high number of individuals affected by chronic wounds, the development of safe and effective wound care treatments has become a key area of research in biology. Wound healing is a complex, multistage process that involves hemostasis, inflammation, angiogenesis, fibroblast proliferation, collagen formation, and tissue remodeling. During skin wound healing, reepithelization involves keratinocyte proliferation and migration. It is widely recognized that plant-derived extracts and their natural compounds exhibit strong efficacy in wound management, targeting various aspects such as inhibiting pro-inflammatory cytokine production, decreasing oxidative stress, boosting antioxidant enzyme activity, and promoting neovascularization and angiogenesis (Figure 4) [95]. Multiple preclinical studies have shown that plant-derived products can effectively influence the proliferation and differentiation of mesenchymal stem cells. Two independent studies demonstrated that wheat- and grapefruit-derived PDEs promote skin regeneration by stimulating the proliferation and migration of human epidermal keratinocytes (HaCaT cells) in a dose-dependent fashion. Both PDEs were also able to increase the tube-like structure formation in human umbilical vein endothelial cells (HUVEC), suggesting that these PDEs were proangiogenic and had the ability to induce vascular formation during wound healing. Moreover, both studies also reported that the wheat-derived and grapefruit-derived PDEs were able to increase the mRNA levels of collagen type I (COL1A1) in human dermal fibroblast (HDF) and HaCaT cells. Further investigations also showed that the grapefruit-derived PDEs induced the upregulation of the expression of various wound-healing factors, including laminin, fibronectin, vimentin and epidermal growth. Collectively, these results suggest that both wheat-derived and grapefruit-derived PDEs promote skin regeneration by enhancing both wound healing and wound closure (Figure 4) [96,97]. Kim et al. investigated the wound healing and antioxidant effect of EVs derived from Aloe vera peels (AVDVs) on human keratinocytes (HaCaT) and human dermal fibroblasts (HDF). Multiple studies have demonstrated that the Aloe vera contains a variety of phenolic compounds, such as cinnamic acids, chromones, anthracenes, and flavonoids. This study showed that AVDVs displayed good compatibility on human skin cells and were internalized into HaCaT cells via clathrin-, caveolae-mediated endocytosis, and membrane fusion. A scratch assay showed that AVDVs promotes the migration ability of HaCaT and HDF. Again, AVDVs significantly upregulated the mRNA expression of Nrf2, HO-1, CAT, and SOD genes in H2O2-treated HaCaT cells. Their findings revealed that AVDVs could activate the antioxidant defense mechanisms and wound healing process via the Nrf2 activation [98]. Ginger-derived nanovesicles, which contain shoal and gingerol, have been shown to suppress the expression of secreted hemotoxic proteins and modulate the levels of key mitochondrial and cytoplasmic proteins, including heat shock proteins, axin, and kinesin—factors that play a significant role in intestinal wound healing [30]. Aloe Saponaria is recognized for its content of flavonoids and phenolic acid compounds, which possess antioxidant and anti-inflammatory properties. An interesting study demonstrated that EVs from Aloe Saponaria (ASDVs) showed a therapeutic activity in treating chronic wounds. Results indicated that ASDVs exhibited no significant cytotoxicity across various cell types despite high intracellular uptake. However, when applied to lipopolysaccharide (LPS)-stimulated RAW264.7 macrophages, ASDVs led to significant reductions in pro-inflammatory gene expression, including IL-6 and IL-1β. Additionally, ASDVs were found to promote proliferation and migration of HDF and enhance tube formation in HUVEC cells, suggesting a stimulatory effect on angiogenesis, a critical aspect of effective wound healing [99]. Wound healing properties of ginger-derived vesicles (GDVs) have been studied in vitro and in vivo in the context of colon inflammation. It is shown that GDVs contain high levels of lipids, few proteins, microRNAs (miRNAs), and large amounts of ginger bioactive constituents (6-gingerol and 6-shogaol). They showed that GDVs are predominantly absorbed by intestinal epithelial cells (IECs) and macrophages and are non-toxic. Using different mouse colitis models, they showed that GDVs reduce acute colitis, enhance intestinal repair, and prevent chronic colitis and colitis-associated cancer. It is also noted that the oral administration of GDVs increases the survival and proliferation of IECs and reduces the pro-inflammatory cytokines (TNF-α, IL-6, and IL-1β), and increases the anti-inflammatory cytokines (IL-10 and IL-22) in colitis models, suggesting that GDVs have the potential to reduce damaging factors while promoting the healing effect [74]. Finally, a recent study of EVs isolated from Opuntia ficus-indica fruit (OFI-EVs) offers a further potential candidate for healing chronic cutaneous wounds. The findings showed that pretreatment with OFI-EVs reduced the activity and gene expression of pro-inflammatory cytokines (IL-6, IL-8, and TNF-α) in the LPS-stimulated human leukemia monocytic cell line (THP-1). Furthermore, OFI-EVs promote the migration of HDFs, speeding up the normal wound healing processes [100].

## 4. PDEs as Therapeutic Delivery Agents

Compared to traditional drug therapies, nanomedicine delivery systems offer several key advantages, including enhanced drug protection, targeted delivery, increased stability, and improved solubility, the ability to cross biological barriers, and reduced dosage and toxicity [101]. PDEs stand out as natural drug delivery systems, providing distinct benefits such as the fact that PDEs, with their natural lipid bilayer vesicle-like structure, function as drug delivery tools without requiring extensive preparation processes. PDEs demonstrate inherent targeting potential due to the differential molecular composition of their lipid bilayers. Again, PDEs exhibit favorable biocompatibility, triggering minimal immune reactions or toxic side effects [102]. For instance, cabbage-derived EVs showed no significant decrease in cell survival when added to human and mouse cells over 72 h; instead, they promoted cell proliferation [103]. Similarly, strawberry-derived EVs were readily taken up by human bone marrow mesenchymal stem cells without inducing any toxicity, and they may also contribute to shielding the cells from oxidative stress. Notably, PDEs have not been associated with adverse effects, unlike synthetic or animal-derived EVs. Additionally, when exosomes were intravenously administered to pregnant mice, they did not cross the placental barrier, further demonstrating their safety [58,104]. Finally, plants are a renewable resource, and extracting vesicles from them is a simple and sustainable process. This makes PDEs an environmentally friendly option for drug delivery. Given these advantages, PDEs are gaining significant attention as promising nanocarriers, particularly for their ability to easily cross mammalian barriers. Current research suggests that PDEs can directly impact recipient cells by transferring their internal components. Several studies indicate that PDEs can be used to deliver medicinal agents, positioning them as promising nanovectors for future drug delivery applications [35,105]. The development of targeted drug delivery mechanisms is crucial for improving treatment outcomes across a range of diseases, and PDEs offer a novel and efficient approach. Several reviews have already highlighted the numerous advantages of PDEs as effective drug delivery tools, underscoring their potential for future therapeutic applications.

## 5. Loading Strategies of PDEs as Drug Carriers

**Figure 5:**
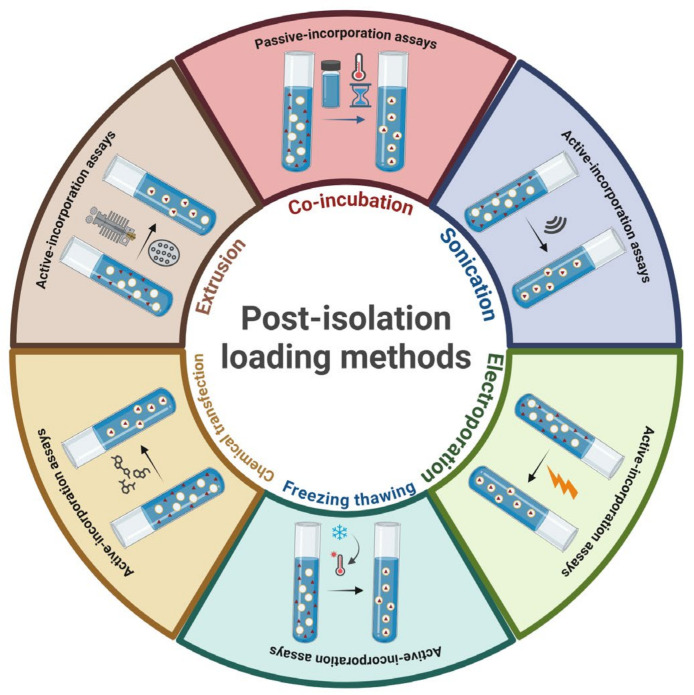
Active and passive loading methods. Drugs, include nucleic acid as (mRNA, small non-coding RNA) chemotherapy agents, and small-molecule drugs, which can be loaded into PDEs using these methods. Created by BioRender scientific illustration software.

In vitro enzymatic digestion investigations have demonstrated the stability of EVs in simulated gastric and/or intestinal fluids, maintaining their physicochemical properties such as size, size distribution, and surface charge. This indicates their possible application as delivery vehicles, particularly for oral drug administration. The functionality of EVs and their cellular uptake are both affected by their lipid content and the arrangement of their structural components [106]. Notably, PA, recognized for its significance in intercellular communication and its crucial role in drug delivery, stands out as one of the most abundant lipid molecules present in PDEs. EVs can be engineered to carry external therapeutic agents—including proteins, miRNAs, siRNAs, and expression vectors—to enhance their effectiveness in disease treatment. Additionally, they are used in nutraceutical and cosmetic applications to boost the activity of naturally occurring bioactive compounds [105]. As illustrated in Figure 5, drug encapsulation within EVs generally follows two primary strategies: passive and active cargo loading.

### 5.1. Passive Cargo Loading

The passive drug loading method involves incubating PDEs at a specific temperature. During this process, EVs are exposed to drugs, allowing them to permeate into the exosomes driven by a concentration gradient. This approach depends on the interplay of diffusion, hydrophobic/hydrophilic properties, and electrostatic interactions between the drug molecules and the lipid bilayer of the vesicles [107]. The efficiency with which drug molecules are loaded into vesicles largely depends on their hydrophobic properties. Hydrophobic drugs tend to associate with the lipid bilayer of the vesicle membrane, becoming embedded within it, whereas hydrophilic drugs are typically enclosed within the vesicles’ internal aqueous core [108]. Most EVs typically exhibit a negative surface charge, which can be advantageous for both active and passive cargo loading techniques. This charge property facilitates the attraction of positively charged drugs, such as doxorubicin, aiding in their incorporation into the vesicles [109]. Furthermore, EVs are commonly used to encapsulate negatively charged molecules such as nucleic acids (RNAs, microRNAs, and DNAs), as well as neutral compounds like curcumin and folic acid. In the case of these compounds, their lipophilic properties enable encapsulation by overcoming adverse electrostatic interactions. Certain natural compounds offer considerable health benefits but are hindered by poor solubility and bioavailability, which restrict their effectiveness [110]. Curcumin, as an example, is commonly employed as a prototype hydrophobic compound for the purpose of encapsulation. Although curcumin possesses a wide range of bioactive and therapeutic properties—such as anti-inflammatory, anti-cancer, and antioxidant effects—its hydrophobic character and strong affinity for lipid membranes limit its solubility and reduce its effectiveness in clinical applications [111]. Various technologies have been developed to enhance curcumin bioavailability, including encapsulation in liposomes, polymeric nanoparticles, biodegradable microspheres, cyclodextrin, hydrogels, and more recently, mammalian exosomes. In a study conducted by Vashisht et al., curcumin was passively loaded into milk-derived exosomes through overnight incubation, achieving a loading efficiency of 70.46%. The stability of free curcumin versus exosome-encapsulated curcumin was assessed using spectrophotometric analysis in PBS and simulated digestive conditions. The results showed that curcumin encapsulated within exosomes exhibited significantly improved solubility and stability compared to its free form. Free curcumin was found to degrade rapidly during in vitro digestion, whereas exosome-encapsulated curcumin maintained its structural integrity under various stressors, including fluctuating temperatures, extreme pH levels, and enzymatic activity [112]. In a separate investigation, Sun et al. examined the enhanced anti-inflammatory effects of curcumin encapsulated in EL-4-derived exosomes (originating from a murine tumor cell line) on activated myeloid cells in vivo. Their findings revealed that curcumin delivered via extracellular vesicles, remained more stable, and achieved higher systemic concentrations than free curcumin [113]. Together, these studies suggest that mammalian-derived EVs—such as exosomes from intestinal epithelial cells or outer membrane vesicles (OMVs) from gut microbiota—may naturally enhance the absorption and bioavailability of hydrophobic, poorly water-soluble dietary compounds. This increased cellular uptake may, in turn, amplify their positive effects on health. An alternative to passive loading involves incubating donor cells with the bioactive compound of interest; the cells then internalize and package the compound into EVs during secretion, effectively producing vesicles pre-loaded with the desired therapeutic agent [114].

### 5.2. Active Cargo Loading

Active cargo loading involves methods that temporarily compromise the integrity of EV membranes, allowing target compounds to diffuse into the vesicles during this transient disruption. Common techniques include sonication, extrusion, and freeze–thaw cycles, all of which are employed to permeabilize the membrane structure [115]. Following loading, the EV membrane can be restored to its original form. In a study by Xin Luan et al., it was found that exosome membranes regained their structural integrity within one hour of incubation at 37 °C. Nevertheless, there are concerns that these membrane-disrupting methods could potentially impair the structural stability and targeting functionality of EVs. Despite such concerns, active loading methods present a key advantage: they offer significantly higher loading efficiency compared to passive techniques, while still being relatively straightforward to perform [116]. For instance, Fuhrmann et al. applied sonication to disrupt EV membranes and reported up to an 11-fold increase in the loading efficiency of hydrophilic porphyrins compared to passive loading approaches [116]. There is growing interest in incorporating miRNAs, siRNAs, and DNAs into EVs for use in gene therapy. Among the available methods, electroporation is the most used technique for actively loading nucleic acids into EVs. This process involves applying an electric field to exosomes suspended in a conductive solution, temporarily creating microscopic pores in their membranes. Electroporation is especially effective for introducing larger molecules like siRNAs or miRNAs, which are unable to passively diffuse through the EV membrane as small hydrophobic molecules can [117]. Plant-derived extracellular vesicles (PDEs) generally range in size from 50 to 500 nm, and maintaining a consistent size distribution is essential for their effectiveness in drug delivery systems. To address this, PDEs can be modified to generate uniformly sized nanoparticles. Lipids derived from PDEs can be used to reconstruct synthetic EVs that are pre-loaded with therapeutic agents, thereby improving their uptake by mammalian cells. While these lipid nanocarriers differ from exosomes in structure and function, they offer the benefits of a natural, food-derived delivery system [118]. Loureiro et al. extracted nanolipids from grape-derived EVs using the “Bligh and Dyer technique”, a liquid–liquid extraction process. These nanolipids were then processed through a 200 nm liposome extruder to obtain uniformly sized nanovectors [119]. Similarly, Zhang et al. applied a re-engineering strategy to incorporate doxorubicin into nanovesicles derived from ginger EVs. Using sonication, lipids isolated from ginger EVs were reorganized into nanostructures, and doxorubicin was loaded through electrostatic interactions. To enhance targeting against colon cancer, the vesicles were functionalized with folic acid, a targeting ligand. This formulation enabled pH-sensitive drug release, optimizing its therapeutic action for colon cancer treatment [20]. In another study, Wang et al. extracted lipids from grapefruit-derived EVs (GF-EVs) and used them to reconstruct artificial vesicles. These vesicles were effectively bound to hydrophobic compounds such as curcumin, folic acid, and Zymosan A while retaining their biological functionality. Additionally, they efficiently delivered biotinylated DNA, proteins, and active siRNA into target cells in vitro [53]. PDEs offer the advantage of being customizable through the attachment of specific bioactive molecules to their surface. Furthermore, drug delivery efficiency of PDEs can be significantly improved by coating them with mammalian-derived membranes containing specific receptors, enhancing their targeting capabilities [105].

### 5.3. PDEs as Carriers for Nucleic Acids Delivery

Many PDEs demonstrate effective loading capacity and show great potential for therapeutic applications with nucleic acid-based drugs. For instance, miRNA was loaded onto cabbage-derived nanovesicles (CBDVs), and real-time PCR analysis showed a 667,000-fold increase in miRNA levels after loading into CBDVs, indicating a high number of miRNAs successfully loaded. After co-cultivating with colon cancer cells for 72 h, miRNA levels increased by over 246,000 times in the cells treated with CBDVs [103]. In a separate study, grapefruit-derived EVs were used to deliver miR-17, a microRNA with known tumor-suppressive properties, for the treatment of brain tumors in mice, effectively slowing tumor progression. Findings indicated that EVs administered through the nasal route could cross the blood–brain barrier, enabling the delivered miR-17 to reach brain tissue and exert its therapeutic effects. This strategy presents a promising non-invasive method for treating neurological disorders via intranasal delivery [120]. Different types of PDEs enable various drug delivery pathways. For instance, nanovesicles derived from Acerola have been successfully used to deliver nucleic acid-based drugs to the digestive tract through oral administration. Their gene-suppressing effect in the small intestine and liver reached its peak one day after administration, demonstrating their potential as an oral drug delivery system (DDS) for nucleic acids in the digestive tract [121]. Further investigation has been carried out by Del Pozo-Acebo’s group. They analyzed the miRNAs content of broccoli-derived exosomes and selected five promising candidates for loading into the same vesicles to increase the amount of these miRNAs to a pharmacologically effective dosage. They employed lipofection to introduce miRNAs into exosomes, leading to a 600-fold enhancement in their expression levels. Their study showed that miRNAs enclosed within broccoli-derived EVs exhibit greater resistance to gastric digestion compared to those that are not encapsulated. Moreover, these vesicles reduced the viability of Caco-2 cells by 30%, suggesting a potential application in reducing the viability of cancer cells [122]. In another study, oral delivery of ginger-derived nanovesicles carrying siRNA-CD98 was found to efficiently and specifically target colon tissue, resulting in decreased CD98 expression. This strategy may enhance the therapeutic potential of these nanovesicles for the treatment of ulcerative colitis [123] (Table 1).

### 5.4. PDEs as Carriers for Chemotherapy Drug Delivery

Several existing drugs have been loaded into PDEs and studied for their potential in treating tumor cells. For instance, Sorafenib is an oral treatment for hepatocellular carcinoma (HCC), and its low water solubility, harsh gastrointestinal environment, and off-target effects contribute to its poor bioavailability. A recent study demonstrated that Sorafenib was encapsulated in kiwifruit-derived vesicles (KEVs). Data showed that encapsulating Sorafenib in KEVs reduced its leakage in the gastrointestinal environment and improved its ability to cross the epithelium cells. It was revealed that KEVs-Sorafenib (KEVs-SFB) could maintain their integrity in the gastrointestinal environment and enter systemic circulation. To assess the biocompatibility of KEVs-SFB, their hemolytic activity in rat serum was examined, showing no significant hemolysis, even at high concentrations. Additionally, KEVs-Sorafenib demonstrated anti-tumor activity in Hep-G2 cells [126]. In another interesting study, doxorubicin was loaded into cabbage-derived exosomes, revealing that these nanoparticles can successfully reach the tumor site and inhibit the proliferation of colon cancer cells. Their inherent capacity to reduce inflammation and inhibit apoptosis further boosts their potential as effective drug delivery systems in cancer treatment [103]. In line with this study, another group encapsulated doxorubicin in celery-derived exosomes and investigated its anti-tumor effects. Interestingly, exosomes derived from celery showed higher absorption efficiency and greater therapeutic effectiveness for doxorubicin than liposomes. This high cellular uptake efficiency is attributed to the higher proportion of diacylglycerol (DAG) and PA in the lipid composition of the exosomes. Furthermore, exosome vesicles derived from celery and loaded with doxorubicin demonstrated a greater reduction in tumor mass in mice compared to the liposome–doxorubicin system. Toxicity assessments in mice following intravenous administration showed no signs of deteriorating health, with blood and organ analyses confirming no toxicity from celery EVs. This study also demonstrated that EVs can persist for extended periods, increasing their chances of efficiently reaching target sites [127]. A separate study highlights the potential synergy between the natural components of PDEs and conventional drugs. Bitter melon-derived exosomes have demonstrated both anti-tumor and anti-inflammatory effects. Their anti-inflammatory properties enhance the effectiveness of 5-fluorouracil (5-FU) and help reduce drug resistance in the treatment of oral squamous cell carcinoma (OSCC). Previous research by this group has shown that the activation of the NOD-, LRR-, and pyrin domain-containing protein 3 (NLRP3) protein plays a significant role in the resistance of OSCC cells to 5-FU. Interestingly, bitter melon exosomes (BMEVs) were found to significantly lower the expression of NLRP3. Additionally, the RNAs present in BMEVs contribute to their anti-inflammatory properties, enabling them to enhance the therapeutic efficacy of 5-FU against OSCC in a synergistic manner, both under in vitro and in vivo conditions [128]. Zhang and colleagues showed that ginger-derived vesicles (GDVs) loaded with doxorubicin can be effectively ingested by intestinal cancer cells. Their results revealed that GDVs could load doxorubicin with high efficiency and exhibited a favorable pH-dependent drug release profile. They also showed that doxorubicin-loaded GDVs successfully suppressed tumor growth in a Colon-26 xenograft mouse model [20]. Another group demonstrated that the human recombinant protein HSP70 can be loaded into tomato and grapefruit EVs through a series of passive and active loading processes. HSP70 helps to protect cells from stress-induced damage and can enhance the sensitivity of tumor cells to chemotherapy. Additionally, these EVs possess antioxidant properties due to their active biomolecules, which include flavonoids, ascorbic acid, and anthocyanins [129] (Table 2).

### 5.5. PDEs as Carriers for Small Molecule Chemical Drug Delivery

PDEs are orally administrable and exhibit distinct cellular targeting profiles based on their source of origin. For example, nanovesicles derived from garlic chives demonstrated inherent anti-inflammatory properties. When the anti-inflammatory drug dexamethasone was encapsulated within these nanovesicles, inflammation in microglia cells could be further alleviated [18]. Yang and colleagues conducted a study demonstrating the effectiveness of 6-shogaol encapsulated in ginger-derived nanoparticles for treating ulcerative colitis. They observed that 6-shogaol showed enhanced release kinetics after 24 h when compared to its free (unencapsulated) form. The nanoparticles exhibited strong internalization by macrophages, with maximum uptake occurring after 3 h. When assessing the bioavailability of both the free and encapsulated drug, they observed a significantly higher maximum concentration and retention of the drug in the colon following oral administration of the nanoparticle-encapsulated 6-shogaol. These results suggest enhanced efficacy in promoting intestinal mucosal healing [133]. Grapefruit-derived EVs possess multiple features that make them well-suited for use in oral drug delivery systems, such as biocompatibility, biodegradability, stability under different pH conditions, and cell-specific targeting capabilities. When paired with the anti-inflammatory drug methotrexate (MTX), these EVs loaded with MTX demonstrated lower toxicity than free MTX and enhanced therapeutic effectiveness in a mouse model of DSS-induced colitis. This suggests that grapefruit-derived EVs could serve as intestinal immune-modulators and be developed for oral delivery of small-molecule drugs to mitigate inflammation in human diseases [53]. PDEs not only have good absorption in the intestine but also deepen the penetration depth of drugs on the skin surface and greatly increase the amounts of drugs absorbed by the cortex. Skin absorption is feasible because exosomes can traverse transcellular and intercellular routes through lipid fusion mechanisms, including phagocytosis, micropinocytosis, clathrin-mediated endocytosis, and even via hair follicles. Abraham and coworkers suggested that plant crystal EVs and classical EVs extracted from cucumber could be used as nanocarriers for transdermal applications. The study found that a lipophilic active ingredient was twice as effective at penetrating the skin when applied in combination with EVs as in a buffer solution. This increased efficiency was observed in both the EVs prepared using the classical method and in plant crystal EVs [134] (Table 3).

## 6. PDEs Surface-Modified for Drug Delivery System

PDEs, which possess inherent therapeutic properties and can facilitate intercellular communication by transporting functional cargoes to receptor cells, are a promising new therapy. However, their use is limited by challenges such as low targeting efficiency and poor homogeneity. To address these issues, various advanced technologies have been developed to enhance the functionality of PDEs, improve their ability to bind to specific target cells, and expand their therapeutic potential. One of these strategies is surface modification. Surface modification, through methods like coupling reactions or lipid assembly strategies, allows for the attachment of peptides, proteins, targeting molecules (e.g., folic acid), or compounds (e.g., azide derivatives) to the vesicle surface, adding new functionalities while maintaining vesicle integrity. This can lead to improved targeting, reduced off-target effects, enhanced bioavailability, imaging capabilities, and photothermal effects. Furthermore, modifying PDEs with polyethylene glycol (PEG)-lipid insertion can extend their circulation time in the bloodstream, increase stability, and facilitate delivery to tumor tissues via enhanced permeability and retention effects [136]. For instance, Niu and colleagues developed an innovative system combining small heparin and Niu and colleagues designed a novel delivery platform by integrating small heparin nanoparticles loaded with doxorubicin into grapefruit-derived exosomes. MTT assays across various cell lines, along with histological evaluations of mouse organs, confirmed the absence of toxicity following intravenous administration. The amino groups present in the lipid membrane of the exosomes interacted with the carboxyl groups of heparin, creating a three-dimensional network that enhanced the doxorubicin loading capacity by four times. Additionally, the incorporation of heparin nanoparticles extended the circulation time of the grapefruit-derived EVs, thereby increasing their likelihood of crossing the blood–brain barrier and targeting glioma tissues. The study showed that these EVs entered glioma cells through receptor-mediated transcytosis and membrane fusion, enabling efficient delivery of antiproliferative agents to tumors within the central nervous system [137].

Supporting this approach, another study modified the surface of lemon-derived EVs with heparin-cRGD, a cyclic peptide known for its tumor-targeting properties. When these engineered vesicles were incubated with doxorubicin, they exhibited promising results in the treatment of ovarian cancer. Specifically, the drug-loaded, modified EVs demonstrated superior anti-tumor and anti-proliferative activity compared to free doxorubicin, mainly due to enhanced cellular uptake. This system also showed excellent biocompatibility and safety [130]. Researchers have greatly enhanced the targeting efficiency of cells with folate receptors by co-delivering therapeutic agents using grapefruit-derived EVs and folic acid. They demonstrated the in vivo targeting specificity of grapefruit-derived nanovectors in two tumor xenograft models—the mouse CT26 colon cancer model and the human SW620 colon cancer SCID mouse model—by co-delivering therapeutic agents with folic acid, resulting in a significant increase in affinity for folate receptor–expressing cells [102]. In another investigation. Zhang and his team utilized EVs derived from ginger as a delivery vehicle for doxorubicin. To enhance the EVs’ efficacy, they functionalized their membranes with folic acid, targeting tumor cells that express folate receptors. Additionally, they assessed the cytotoxicity of the ginger-derived EVs using various assays, such as MTT and electric cell-substrate impedance sensing. The results demonstrated that the ginger-derived EVs had better safety compared to a well-established cationic liposome [20].

## 7. Conclusions and Prospective

The use of plants as a source of PDEs offers a promising alternative to mammal-derived EVs and synthetic biomaterials such as lipid-like nanoparticles, biodegradable polymers, and hydrogels. PDEs may address key concerns relating to the usage of synthetic materials like delivery systems such as immunogenicity, toxicity, and scalability. Since plants are regularly consumed as part of the human diet, PDEs are inherently biocompatible and safe, minimizing the risk of adverse immune reactions. In contrast to synthetic nanoparticles—which require individual safety assessments for each component—PDEs are naturally non-toxic and organically derived. Moreover, their production is more cost-effective and sustainable, with the possibility of utilizing agricultural by-products or edible plant materials, thereby overcoming the scalability limitations of mammalian EVs and synthetic systems. Importantly, PDEs are not just inert carriers; they inherently contain bioactive compounds such as plant RNAs, lipids, and proteins that contribute to intercellular communication and regulation of gene expression in physiological and pathological conditions. These intrinsic therapeutic properties, combined with their safety profile, cost efficiency, and natural origin, position PDEs as a powerful and versatile platform for future biomedical applications, particularly in drug delivery, immune modulation, and chronic disease management. These EVs also carry bioactive compounds like antioxidants and anti-inflammatory agents, which are particularly valuable for pharmaceutical applications, as they can work synergistically with external drugs. The potential applications of PDEs are vast, from delivering natural supplements and micronutrients to supporting therapies for conditions such as cancer, inflammatory diseases, and skin disorders. They also offer potential as natural carriers for therapeutic agents due to their easy accessibility and cost-effectiveness, without the need for chemical solvents during isolation. Although PDEs have been implicated in beneficial inter-organismal communication, our understanding of their roles remains limited. Notably, the dual functionality of vesicle-mediated interactions—originating from both plant hosts and pathogens—remains poorly characterized. Further studies are needed to elucidate the underlying mechanisms and bidirectional dynamics of these vesicular exchanges, particularly in the context of plant–microbe interactions and their potential effects on human health.

Comprehensive research into the pharmacological and targeted delivery properties of PDEs is necessary to fully define their therapeutic potential. Establishing a database of their protein content would help identify specific markers for characterization, while data on their nucleic acids and secondary metabolites could aid in virtual screening. A thorough understanding of the mechanisms behind their cellular uptake and bio-distribution is critical for utilizing EVs effectively in targeting specific therapeutic sites, such as tumors, the intestines, or the central nervous system.

As a result, ongoing research is increasingly focused on modifying the surface of EVs to achieve precise targeting. Leveraging this valuable resource could lead to significant innovations in drug delivery, including the development of systems that can enhance the efficacy of medications by interacting synergistically with the natural components of EVs. This has the potential to advance the creation of vaccines, supplements, and treatments for conditions that are currently inadequately addressed by existing therapies, particularly those related to rapid clearance of active ingredients or poor solubility in biological environments.

## Figures and Tables

**Table 1:** PDEs as carriers for nucleic acid delivery.

Source	Compound/Molecule	Mode of Action	Reference
Grapefruit	MiR-17	Tumor-inhibiting effects, used to treat brain tumors in mice and reduce the growth rate of tumors.	[120]
Acerola	MiR-340 and miR-146	Orally delivered miRNA/vesicles reduced the expression of miRNA’s target gene.	[121]
Ginger	siRNA-CD98	Orally administered siRNA-CD98/GDLVs specifically targeted colon tissues and effectively decreased CD98 expression.	[123]
Grapefruit	MiR-18	Grapefruit-derived lipids delivering miR18 prevent liver metastasis by promoting the activation of M1 macrophages.	[124]
Ginger	SiRNA survivin	Suppressed tumor growth in a mouse xenograft model.	[125]

**Table 2:** PDEs as carriers for chemotherapy drug delivery.

Source	Compound/Molecule	Mode of Action	Reference
Kiwi	Sorafenib	Sorafenib showed anti-tumor effects in Hep-G2 cells.	[126]
Cabbage	Doxorubicin	Suppressed the growth of colon cancer cells.	[103]
Celery	Doxorubicin	Celery-derived vesicles loading with doxorubicin decreased tumor size in mice.	[127]
Bitter melon	5-FU	Exosomes derived from bitter melon loaded with 5-FU exhibited a synergistic therapeutic effect against OSCC in both in vitro and in vivo settings.	[128]
Ginger	Doxorubicin	Doxorubicin effectively suppressed tumor growth in a Colon-26 xenograft tumor model.	[20]
Lemon	Doxorubicin	Lemon vesicles containing doxorubicin effectively penetrated doxorubicin-resistant cells, and produced a strong anti-tumor effect.	[130]
Aloe vera gel	Doxorubicin and indocyanine	Demonstrated strong targeting abilities for breast tumors in both in vitro and in vivo settings.	[131]
Grapefruit	Doxorubicin and curcumin	In vivo experiments demonstrated a significant inhibition of breast tumor growth in mice.	[132]

**Table 3:** PDEs as carriers for small molecule chemical drug delivery.

Source	Compound/Molecule	Mode of Action	Reference
Ginger	6-shogaol	Improved effectiveness in supporting the healing of intestinal mucosa.	[133]
Grapefruit	methotrexate	Increased therapeutic effectiveness in mice with DSS-induced colitis.	[53]
	HSP70 and BSA	Grapefruit vesicles enhanced the absorption of exogenous proteins (HSP70 and BSA) in both, in vitro and in vivo.	[135]
	anti-Stat3 inhibitor (JSI-124)	Decreased Stat 3 expression in mice treated intranasal with grapefruit vesicles containing JSI-124.	[53]
	inflammatory chemokine receptor	Grapefruit-derived nanovectors coated with membranes enriched in inflammatory-related receptors from activated leukocytes (IGNVs) have improved targeting capabilities for inflammatory tumor tissues.	[132]
Aloe	Indocyanine green	Vesicles containing indocyanine green suppressed melanoma growth.	[57]
